# CTpathway: a CrossTalk-based pathway enrichment analysis method for cancer research

**DOI:** 10.1186/s13073-022-01119-6

**Published:** 2022-10-13

**Authors:** Haizhou Liu, Mengqin Yuan, Ramkrishna Mitra, Xu Zhou, Min Long, Wanyue Lei, Shunheng Zhou, Yu-e Huang, Fei Hou, Christine M. Eischen, Wei Jiang

**Affiliations:** 1 Department of Biomedical Engineering, Nanjing University of Aeronautics and Astronautics, No. 29, Jiangjun Avenue, Nanjing, 211106 Jiangsu Province China; 2 Department of Pharmacology, Physiology, and Cancer Biology, Sidney Kimmel Cancer Center, Thomas Jefferson University, 233 South 10th St., Philadelphia, PA 19107 USA

**Keywords:** Pathway enrichment analysis, Pathway crosstalk, Risk pathway, Molecular interaction, Network analysis

## Abstract

**Background:**

Pathway enrichment analysis (PEA) is a common method for exploring functions of hundreds of genes and identifying disease-risk pathways. Moreover, different pathways exert their functions through crosstalk. However, existing PEA methods do not sufficiently integrate essential pathway features, including pathway crosstalk, molecular interactions, and network topologies, resulting in many risk pathways that remain uninvestigated.

**Methods:**

To overcome these limitations, we develop a new crosstalk-based PEA method, CTpathway, based on a global pathway crosstalk map (GPCM) with >440,000 edges by combing pathways from eight resources, transcription factor-gene regulations, and large-scale protein-protein interactions. Integrating gene differential expression and crosstalk effects in GPCM, we assign a risk score to genes in the GPCM and identify risk pathways enriched with the risk genes.

**Results:**

Analysis of >8300 expression profiles covering ten cancer tissues and blood samples indicates that CTpathway outperforms the current state-of-the-art methods in identifying risk pathways with higher accuracy, reproducibility, and speed. CTpathway recapitulates known risk pathways and exclusively identifies several previously unreported critical pathways for individual cancer types. CTpathway also outperforms other methods in identifying risk pathways across all cancer stages, including early-stage cancer with a small number of differentially expressed genes. Moreover, the robust design of CTpathway enables researchers to analyze both bulk and single-cell RNA-seq profiles to predict both cancer tissue and cell type-specific risk pathways with higher accuracy.

**Conclusions:**

Collectively, CTpathway is a fast, accurate, and stable pathway enrichment analysis method for cancer research that can be used to identify cancer risk pathways. The CTpathway interactive web server can be accessed here http://www.jianglab.cn/CTpathway/. The stand-alone program can be accessed here https://github.com/Bioccjw/CTpathway.

**Supplementary Information:**

The online version contains supplementary material available at 10.1186/s13073-022-01119-6.

## Background

Over 15 years, significant efforts have been made to annotate the functions of individual genes and construct higher order functional knowledgebases, such as the Kyoto Encyclopedia of Genes and Genomes (KEGG) and Gene Ontology (GO) [1, 2]. However, it still remains quite challenging to systematically interpret biological meaning from the expression changes of thousands of genes in a specific model system, such as disease versus control. To determine this, a routinely used method is the screening of differentially expressed genes (DEGs) followed by pathway enrichment analysis (PEA).

PEA methods could be categorized into four generations [3]. First-generation methods (e.g., DAVID [4], WebGestalt [5], and several others [6–8]) usually conduct over-representation analysis (ORA) using a hypergeometric or Fisher’s exact test to assess whether the number of input DEGs is significantly higher than that of the genes expected by chance. However, these ORA methods have several limitations. Based on an arbitrary threshold, these methods only select the DEGs that have large expression fold changes (*FC*) or significant *P*-values and treat each selected gene equally. Consequently, these methods achieve highly inconsistent results with small changes (e.g., 1.5 *FC* versus 2.0 *FC*) in thresholds.

To address these limitations, the second-generation methods called functional class scoring (FCS) were developed [9]. FCS methods hypothesize that even though changes of individual genes are small in magnitude, their coordinated expression changes may have a greater impact in modulating a pathway/gene set [10, 11]. A well-known FCS method is the gene set enrichment analysis (GSEA) [11]. GSEA first ranks genes by differential expression *FC*. Enrichment scores (*ES*s) are then calculated for predefined gene sets (pathways or functional gene sets) by considering how well the gene sets are enriched at the top or bottom of the ranked gene lists, which indicate their activation or repression, respectively. Therefore, FCS methods address the limitations of ORA methods.

Previous studies hypothesized that genes with different topological properties have different weights for the linked pathways [12–14]. Because topology information of pathways was used, pathway topology-based (PT) approaches were demonstrated to perform better than the previous approaches and regarded as the third generation of PEA methods [15]. The method CePa calculated the weight of a pathway node based on the network centralities [13]. SPIA considered the influence of the neighboring nodes [14]. TPEA integrated the global upstream/downstream positions and the degrees of all nodes in pathways [12]. A significant drawback of PT methods is that they analyze pathways independently and neglect pathway crosstalk, a common and critical event in biology and disease development.

From the perspective of systems biology, genes may iteratively affect many other genes that exist in multiple pathways, causing pathway crosstalk that accounts for the phenotypes, such as crosstalk between ERK and WNT signaling in tumorigenesis [16]. The latest generation of the PEA method is network topology-based (NT) approaches, which consider pathway crosstalk systematically in a network, such as latent pathway identification analysis (LPIA) [17] and pathways based on network information (PathNet) [18]. LPIA regarded each pathway as one node to construct an edge-weighted pathway network based on shared GO functions and DEGs. LPIA identified pathways by random walk algorithm according to network topology. Although LPIA considered pathway crosstalk, it ignores internal topology property within the pathway. PathNet integrated direct evidence (gene differential expression) and indirect evidence (neighbor gene differential expression), which considered gene interactions in both inter- and intra-pathway, as combined evidence for genes to assess their impacts on pathways. However, the gene interactions only depended on directed neighbors in the pathway network, and it ignored the impact of other genes.

As more biological knowledge was gained, protein-protein interaction (PPI) network and gene expression data were used to detect crosstalk between pathways [19, 20]. Recently, by integrating pathway information, PPI network and gene expression data, Kelder et al. identified indirect associations between pathways in insulin-resistant mouse liver [21]. However, in addition to PPI, transcription factor (TF) regulations also provide additional valuable information about molecular interactions. Additionally, these methods performed enrichment analysis mostly based on KEGG or GO, while numerous high-quality pathways or functional gene sets were also publicly available, such as Reactome [22], PANTHER [23], HumanCyc [24], INOH [25], NetPath [26], PID [27], and WikiPathways [28]. Moreover, NT methods consume more time and more space because of their complexity. For example, LPIA may consume several hours for one test. Therefore, although commonly and widely used, current PEA methods have significant limitations, posing barriers to discovery.

**Fig. 1:**
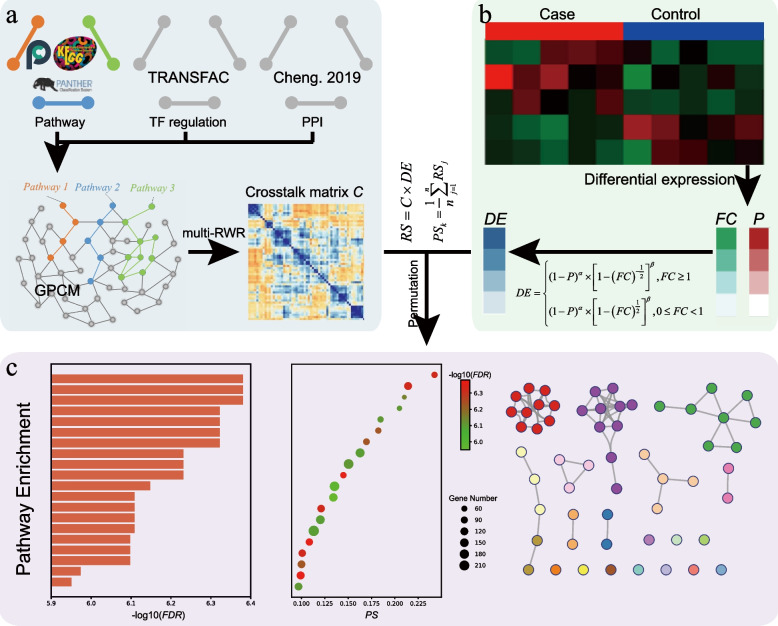
The workflow diagram for CTpathway. **a** GPCM and crosstalk effect matrix construction. GPCM was constructed by integrating pathway, TF regulation, and PPI information. Then, we evaluated the crosstalk effects in GPCM by applying the multi-RWR algorithm to calculate a crosstalk effect matrix *C*. **b** Gene differential expression (*DE*) score calculation. We integrated *FC* and *P*-value to calculate *DE* scores. Next, risk score (*RS*) for each gene and pathway score (*PS*) for each pathway were calculated to identify the significant risk pathways. **c** Visualization of significant pathways by bar graph, bubble plot, and enrichment map

In this study, we provided a new NT method for gene enrichment analysis called CTpathway: a crosstalk-based PEA method in a global pathway crosstalk map (GPCM) (Fig. 1). To obtain better speed, our method was optimized for running time to less than 1 min. Compared with existing methods, including DAVID, GSEA, TPEA, PathNet, and LPIA, CTpathway outperformed in terms of accuracy, robustness, and running time. In addition, CTpathway identified several important cancer pathways, which were not identified by other methods. Furthermore, CTpathway was useful even for data sets with fewer DEGs. By applying CTpathway for several cancer types of different stages (I, II, III, and IV), cancer target pathways were identified in early-stage tissues and blood samples. For breast cancer (BRCA) single-cell RNA-seq (scRNA-seq) data, CTpathway could identify the cell type-related pathways. We also developed an online web tool (http://www.jianglab.cn/CTpathway/) and the stand-alone program (https://github.com/Bioccjw/CTpathway) [29], which allows users to simply upload the gene symbols or entrez gene IDs with log_2_*FC* or *P*-values to identify risk pathways in a specific condition (e.g., disease) by performing the CTpathway method.

## Methods

### Pathway data

We collected eight knowledgebases of human pathways including KEGG [1], PANTHER [23], Reactome [22], HumanCyc [24], INOH [25], NetPath [26], PID [27], and WikiPathways [28]. The interactions of gene and gene products in pathways of Reactome, HumanCyc, INOH, NetPath, PID, and WikiPathways were obtained from Pathway Commons version 10 [30]. Because the information of KEGG and PANTHER in Pathway Commons was not updated, we extracted the interactions in KEGG and PANTHER in March 2019. For KEGG pathways, we downloaded KGML files of 299 pathways and extracted interaction information by iSubpathwayMiner R package [31]. For PANTHER pathways, we downloaded BioPAX files of 138 pathways, and NetPathminer R package [32] was used to extract interaction information. For the other six sources of pathways, we used gene interactions in Pathway Commons. In total, we obtained 375,256 interactions, including 11,556 genes from 2563 pathways involved in eight pathway databases (details in Table 1).

**Table 1:** Summary of data source information in GPCM

Source of interactions	#Pathways	#Genes	#Interactions
KEGG	299	5686	60,576
HumanCyc	238	1658	20,746
INOH	153	939	19,374
NetPath	27	1195	3727
PANTHER	129	2149	26,810
PID	212	2589	21,210
Reactome	1491	9990	266,500
WikiPathways	14	70	97
PPI	-	11,054	79,262
TRANSFAC	-	1947	4657
Total	2563	15,292	442,439

### TF-gene regulation data

We obtained experimentally validated TF-gene regulations from the TRANSFAC Professional database (release: February 2014) [33]. TF-gene regulations, of which at least one node belongs to a pathway, were retained, including 491 TFs, 1614 genes, and 4657 pairs of regulation (Table 1).

### PPI data

We obtained PPIs from 12 sources (Additional file 1: Table S1) collected by previous researchers [34, 35]. To obtain more reliable information, PPIs included in ≥2 sources were retained. Furthermore, we used interactions of which at least one of the interacting nodes belongs to a pathway. Finally, 79,262 PPIs, including 11,054 genes, were used for the next analysis (Table 1).

### Constructing a global pathway crosstalk map (GPCM)

To consider pathway enrichment more systematically, we integrated three kinds of interactions including pathways, PPIs, and TF regulation for constructing a GPCM to simulate natural pathway crosstalk and adding biological knowledge. We used the union of pathway, PPI, and TF-gene information described above. For simplicity here, it was regarded as an undirected network. In total, the network includes 15,292 nodes and 442,439 edges (details in Table 1).

### Gold standard data sets

For comparing CTpathway accuracy with other PEA methods, we used gold standard data sets from the KEGGdzPathwaysGEO R package [36]. It contained 24 data sets involving 12 diseases and 12 target pathways (Additional file 1: Table S2). One disease is corresponding to one target pathway. This data set was widely used as the gold standard for benchmarking in other methods [15, 36, 37]. In addition, to test whether CTpathway could be applied in data sets with fewer DEGs, we analyzed 12 of 24 gold standard data sets with different numbers of DEGs (details in Additional file 1: Table S3).

### Cancer data sets from Gene Expression Omnibus (GEO) and The Cancer Genome Atlas (TCGA) databases

To evaluate reproducibility, we used both microarray data from the GEO database [38] and RNA-seq data from the TCGA database [39] for each of four cancer types (COAD, LIHC, LUAD, and OV). We downloaded eight gene expression data of four cancer types from the GEO database (GSE100179 [40], GSE101685 [41], GSE116959 [42], GSE9891 [43]) and TCGA database (Additional file 1: Table S2). Each data set includes case (cancer) and control (normal) samples (details in Additional file 1: Table S2).

Furthermore, to test whether CTpathway could be applied in early-stage cancer samples, we analyzed ten data sets consisting of different cancer types available in TCGA or GEO (GSE20189; peripheral blood samples of LUAD patients [44]) database. Each data set includes cancer samples of different cancer stages (I, II, III, and IV) and normal samples (details in Additional file 1: Table S2).

To test whether CTpathway could be applied in scRNA-seq data, we downloaded breast cancer (BRCA) scRNA-seq data from the GEO database (GSE118389 [45]) (Additional file 1: Table S2). The BRCA scRNA-seq data contains 1112 cells from six triple-negative breast cancer patients. Here, we used cell type annotation results according to the previous study [45] including B cell, T cell, endothelial cell, epithelial cell, macrophage, and stromal cell. More details about the data sets are shown in Additional file 1: Table S2.

### Differential expression

For the GEO microarray data set, we performed differential expression analysis by R package limma [46] to obtain *FC* and *P*-value. For the TCGA RNA-seq data set, we used R package DESeq2 [47] to obtain *FC* and *P*-value. OV data differential expression profile was from a previous study [48]. For BRCA scRNA-seq data, we performed differential expression analysis between one cell type and the others using function "FindAllMarkers" in R package Seurat V3.2.2 [49]. For some compared methods, which need a set of genes as input, such as DAVID, genes with *FC* > 2 or *FC* < 0.5 and *P*-value < 0.05 were used for functional enrichment analysis. For LPIA, |log_2_*FC*| value was used as differential expression score.

### Gene differential expression score

The *FC* and *P*-value are both important indexes to reflect the differential expression level of genes. Previous studies demonstrated that incorporating *FC* and *P*-value could provide significant improvement to meet the practical needs [50, 51]. We calculated a gene differential expression score to represent the impact of its disrupted expression on pathways. *P*-value ranges from 0 to 1. In order to make the *FC* value between 0 and 1, and keep genes with an *FC* value with *n* and 1/*n* having the same contribution weight, the gene differential expression score was calculated by Eq. (1).

1 $$DE=\left\{\begin{array}{l}{\left(1-P\right)}^{\alpha}\times {\left[1-{(FC)}^{-\frac{1}{2}}\right]}^{\beta }, FC\geq 1\\ {}{\left(1-P\right)}^{\alpha}\times {\left[1-{(FC)}^{\frac{1}{2}}\right]}^{\beta },0\le FC< 1\end{array}\right.$$

When *P*-value (or *FC*) is available or not, *α* (or *β*) equals to 1 or 0. *DE* is the differential expression score, represented as a vector:

2 $$DE=\left[\begin{array}{c}{DE}_1\\ {}{DE}_2\\ {}\vdots \\ {}{DE}_i\\ {}\vdots \\ {}{DE}_L\end{array}\right]$$

where *L* is the intersection number of genes in the expression profile and genes in the GPCM.

### Risk score (RS)

**Fig. 2:**
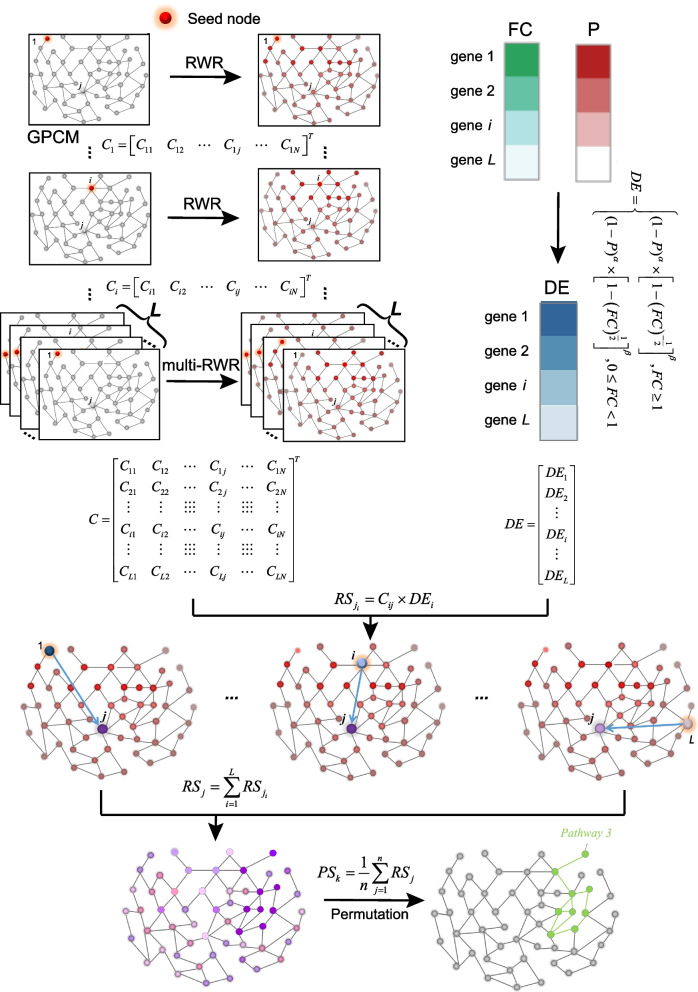
CTpathway algorithm diagram including Multi-RWR. First, gene 1 in the expression profile was taken out as a seed and RWR was used to obtain the crosstalk effect *C*_1_ on all nodes in the network. Next, another gene *i* was chosen to repeat this progress and obtained *C*_*i*_. Finally, we obtained the crosstalk effect matrix *C* after all genes in the profile were taken out as a seed. We also calculated the differential expression (*DE*) score by integrating *FC* and *P*-value. Using both crosstalk and differential expression, we obtained the risk score ${RS}_{j_i}$ of gene *j* impacted by gene *i*. We integrated the risk score of gene *j* impacted by all genes as gene *j* risk score *RS*_*j*_. Finally, we obtained a pathway risk score (*PS*) by averaging all gene risk scores in a pathway and calculated the significance level by permutation

Here in GPCM, for one gene, we calculated a risk score integrating all the nodes (genes) impact on this node (gene). The GPCM was defined as a simple undirected graph *G* = (*V*, *E*), where a *v* ∈ *V* represents a gene and a *e* ∈ *E* represents an edge. First, one gene in the expression file was taken as a seed (i.e., *i*), and given an initial weight score of 1. Then, a random walk with restart (RWR) algorithm [52] was used to simulate the propagation process of crosstalk effect *C*_*i*_ from one to others.

3 $${C}_i^t =r\times W\times{C}_i^{t-1} +\left(1-r\right)\times {N}_i$$

where ${C}_i^1= {N}_i$, *r* is the restart coefficient, *t* is iteration times, *W* was a |*N*| × |*N*| column-normalized adjacent matrix of graph *G*, and *N*_*i*_ is a |*N*| × 1 vector with *i*th element equal to 1 and others all equal to 0. Next, with respect to all the genes in the expression file, we iterated over each gene as a seed. This process was called a multiple random walk with restart (multi-RWR) algorithm (Fig. 2). Here, we measured the magnitude of change between states *t* and *t*-1 as the sum of the absolute difference of the *C*^*t*^ and *C*^*t-*1^. The threshold was set as 10^−10^ to control the iteration times. When it was less than 10^−10^, the iterative computation would stop. Finally, we obtained a crosstalk effect for all genes, named as the crosstalk effect matrix, represented as a matrix:

4 $$C={\left[\begin{array}{cccccc}{C}_{11}& {C}_{12}& \cdots & {C}_{1j}& \cdots & {C}_{1N}\\ {}{C}_{21}& {C}_{22}& \cdots & {C}_{2j}& \cdots & {C}_{2N}\\ {}\vdots & \vdots & \vdots \vdots \vdots & \vdots & \vdots \vdots \vdots & \vdots \\ {}{C}_{i1}& {C}_{i2}& \cdots & {C}_{ij}& \cdots & {C}_{iN}\\ {}\vdots & \vdots & \vdots \vdots \vdots & \vdots & \vdots \vdots \vdots & \vdots \\ {}{C}_{L1}& {C}_{L2}& \cdots & {C}_{Lj}& \cdots & {C}_{LN}\end{array}\right]}^T$$

where *C*_*ij*_ represents the crosstalk effect of gene *j* impacted by gene *i*. Last, we integrated *C* matrix and *DE* vector to calculate the risk score (*RS*) as follows:

5 $$RS=C\times DE$$

For example, we calculated an *RS* of gene *j* impacted by gene *i* as follows:

6 $${RS}_{j_i}={C}_{ij}\times {DE}_i$$

where ${RS}_{j_i}$ represents the risk score of gene *j* impacted by gene *i* and *DE*_*i*_ represents the differential expression score of gene *i*.

For gene *j*, we integrated scores impacted by all genes as the final gene risk score (*RS*_*j*_) as follows:

7 $${RS}_j =\sum\limits_{i=1}^L{RS}_{j_i}$$

where *L* is the intersection number of genes in the expression profile and genes in the GPCM.

### The pathway enrichment score

We obtained *RS* of each gene in GPCM. For a pathway *k*, we calculated a pathway enrichment score *PS*_*k*_ as the average of *RS* values for the genes in pathway *k*. The formula is as follows:

8 $${PS}_k =\frac{1}{n}\sum\limits_{j=1}^n{RS}_j$$

where *n* represents the number of genes in pathway *k*.

### Identification of significant pathways

We performed the permutation analysis to estimate the significance of the pathway. First, we shuffled genes in the differential expression profile. Then, we calculated the pathway enrichment score for each pathway. The background distribution was generated after performing *h* permutations. For a pathway, the empirical *P*-value was defined as the proportion of random pathway enrichment scores (*PS*_*random*_) larger than the real pathway enrichment score (*PS*): *P*-value = ${}^{\left({N}_{PS_{random}> PS}\right)\!}\left/ \!{}_{h}\right.$, where ${N}_{PS_{random}> PS}$ was the number of random pathways that had larger scores than the real pathway. Here, *h* was 1000. However, because of the limited number of permutations, it often produces a *P*-value of 0. To solve this problem, we estimated the exact *P*-value by using the generalized Pareto distribution (GPD) [53]. Because many pathways were involved in this analysis, it was necessary to perform multiple hypothesis testing methods to control the proportion of false positives. We applied the false discovery rate (*FDR*) to account for false positives [54]. The pathways with *FDR* < 0.01 were considered as significant pathways. In addition, CTpathway automatically clusters significant pathways into non-redundant groups. Pairwise similarities between any two significant pathways are computed based on a *Jaccard* similarity coefficient. According to user’s input cutoff of *Jaccard* similarity coefficient, a pathway similarity network is constructed. A default coefficient of 0.3 was set up in this study, which could be customized by the users using our web server. The Markov Cluster (MCL) algorithm [55] was employed to perform clustering process. CTpathway chooses the most significant (lowest *FDR*) pathway within each cluster to represent the cluster. To obtain a better visualization, CTpathway shows the top 20 non-redundant pathways or clusters with low *FDR*, if there are more than 20 clusters or pathways. For each cluster, the top 10 pathways with lower *FDR* are shown in the enrichment map if there are more than 10 pathways which are within one cluster.

### Rank difference (DR) and time difference (DT) values

*DR* value was calculated to represent the rank difference (before and after optimization) of the target pathway as follows:

9 $$DR=\sum\limits_{m=1}^M\frac{\left|{R}_{b_m}-{R}_{a_m}\right|}{K}/M$$

where ${R}_{b_m}$ represents the rank of the target pathway in data set *m* before optimization and ${R}_{a_m}$ represents the rank of the target pathway in data set *m* after optimization. *M* is the number of data sets. *K* is the number of total KEGG pathways. Here, *M* is 24 and *K* is 299.

*DT* value was calculated to represent the running time difference (before and after optimization) as follows:

10 $$DT=\sum\limits_{m=1}^M\frac{T_{b_m}-{T}_{a_m}}{T_{b_m}}/M$$

where ${T}_{b_m}$ represents the running time in data set *m* before the optimization, and ${T}_{a_m}$ represents the running time in data set *m* after the optimization.

### Rank ratio (RR) value

*RR* value was used as the criteria to compare the accuracy of different tools. Each data set *m* had an *RR* value for its target pathway, represented as *RR*_*m*_, which was the rank ratio of the target pathway in data set *m*, and calculated as follows:

11 $${RR}_m=\frac{R_{a_m}}{M}$$

where ${R}_{a_m}$ and *M* were described as above. To make it comparable between different methods, we used KEGG pathways as candidate pathways.

### Stability (S) value

*S* value was used as the criteria to compare the stability or reproducibility of different tools. First, for eight data sets of four cancer types, each of which has microarray data and RNA-seq data, we identified risk pathways by using different methods. For each cancer type, we compared shared significant pathways identified from microarray data and RNA-Seq data. Because compared methods just identified few pathways or no pathways when using routine *FDR* or adjusted *P*-value as a cutoff, pathways with *P*-value < 0.05 were identified as significant pathways for all methods here. *S* value was calculated as follows:

12 $$S=\sum\limits_{d=1}^D\frac{J_d}{D}$$

where *J*_*d*_ represents the *Jaccard* similarity coefficient of different data sets of cancer type *d* and *D* represents the number of all cancer types. In this study, *D* is 4.

### Statistics analysis

Differential expression analysis was performed by R package limma for the GEO data set and R package DESeq2 for the TCGA data set. *P*-value < 0.05 was considered to be statistically significant for DEGs. For CTpathway, permutation analysis was performed to estimate the significance of the pathway; *FDR* < 0.01 was considered as significant. For GSEA, *FDR* < 0.01 was considered as significant. For other compared methods, *P*-value < 0.05 was considered as significant because few pathways or no pathways were identified when using routine *FDR* or adjusted *P*-value as a cutoff.

### Benchmarking

In this study, CTpathway was compared with five widely used tools, including DAVID, GSEA, TPEA, LPIA, and PathNet, in terms of accuracy, reproducibility, and running time. For accuracy, we compared *RR* values for each method using 24 gold standard data sets (Additional file 1: Table S2). For reproducibility, we compared the *S* value calculated for four cancer types (COAD, LIHC, LUAD, and OV) based on different sources (TCGA RNA-seq data and GEO microarray data) of eight gene expression data (Additional file 1: Table S2). For the running time, we used simulated data sets of 500, 1000, 5000, 10,000, and 20,000 genes. Because most of these methods only focused on the pathways defined in KEGG, we used KEGG pathways for comparative analysis when benchmarking.

### Hardware platform

All benchmarks were performed on a computer with 2*Intel Xeon E5-2609 V4 Processor, 2*64G DDR4 RDIMM, 8 DIMM slots, 1*128G SSD 2.5, 1*2TB SATA 3.5, and 2*1080Ti.

### Code availability

CTpathway web server is available at http://www.jianglab.cn/CTpathway/. The CTpathway stand-alone program is available at https://github.com/Bioccjw/CTpathway [29]. Other custom codes used in this study are available from the corresponding authors upon reasonable request.

## Results

### Global pathway crosstalk map (GPCM) and its properties

**Fig. 3:**
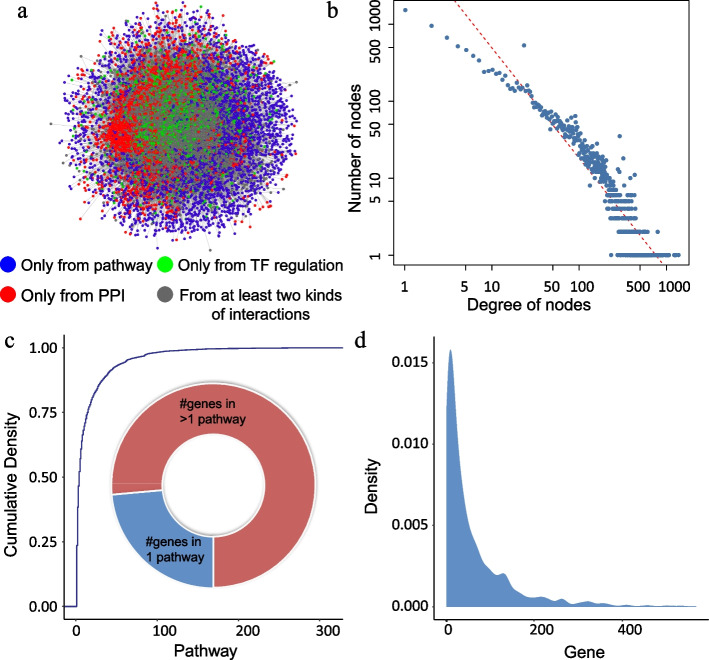
Overview and characteristics of the GPCM. **a** GPCM. Different colors represent different kinds of nodes. Gray dots represent genes from at least two kinds of interactions (pathway, PPI, or TF regulation). **b** The degree distribution of GPCM. **c** The cumulative distribution of the number of genes in pathways. The pie chart shows the proportion of genes in one pathway and in more than one pathway. **d** The density distribution of the number of pathways

By integrating three kinds of interactions including the regulation of TFs to genes from TRANSFAC [33], the PPIs from multiple sources in previous studies [34, 35], and the pathways from eight databases (KEGG [1], Reactome [22], PANTHER [23], HumanCyc [49], INOH [25], NetPath [26], PID [27], and WikiPathways [28]), we constructed a GPCM that included 15,292 nodes and 442,439 edges (Fig. 3a and Table 1). Next, we investigated the topological properties of the GPCM. The degree distribution approximately displayed a power law distribution (Fig. 3b), indicating the network satisfied scale-free topology, a general concept for biological networks. There are some well-known signaling and transcription factor genes with a high degree in the GPCM, such as *EGFR*, *AKT*, *MYC*, and *p53* (Additional file 1: Table S4). The gene with the highest degree in the network is *GNB1*, a subunit of G proteins, which are modulators or transducers in various transmembrane signaling pathways and included in 234 pathways. In addition, we determined that ~75% (*n* > 8800) of the genes participate in more than one pathway (Fig. 3c). Density distribution of pathways showed a positively skewed distribution, which suggested that only a few pathways include a higher number of genes (Fig. 3d). Most of the genes participate in multiple pathways, which suggest that crosstalk exists. Pathway crosstalk was represented by integrating molecular interactions and pathways into a GPCM.

### Crosstalk effect evaluation and pathway identification

Pathways are usually affected by each other in the process of performing functions due to crosstalk [16]. We evaluated the crosstalk effects in GPCM by applying a multi-RWR algorithm to calculate a crosstalk effect matrix, *C*, which exploits the complete network topology (Fig. 2) (details in the “Methods” section). Then, we integrated *FC* and *P*-value as gene differential expression score (*DE*) to reflect the disturbed level of gene expression (details in the “Methods” section). Next, we integrated the *C* matrix and differential expression score (*DE*) to calculate a risk score (*RS*) as the impact of the gene on the pathways (Fig. 2, details in the “Methods” section). For gene *i*, *RS*_*i*_ reflects the risk score of the node *i* in the context of GPCM. We further tested the relationship between *RS* and |log_2_*FC|* based on a lung adenocarcinoma (LUAD) data set (Additional file 1: Table S2) available in the GEO database (GSE116959 [42]). Despite a higher positive correlation (Pearson correlation coefficient *R* ≈ 0.84) (Additional file 2: Fig. S1), we determined several known lung cancer-associated genes with high *RS* and low |log_2_*FC|* (Table 2 and Additional file 2: Fig. S1), such as *TRIM*28, *APP*, *ESR*1, *MYC*, and *EGFR* [56–59]. However, these genes would be overlooked by most of the existing PEA methods because they only consider significant DEGs or high |log_2_*FC|* genes.

**Table 2:** The representative 10 genes with high *RS* and low |log_2_*FC*| value in LUAD (GSE116959)

Gene symbol	EntreZ ID	|log_2_*FC*| value	*DE* value	*RS*	Reference (PMID)
*TRIM*28	10155	0.3854	0.1186	2.3781	33091876
*APP*	351	0.3965	0.1244	1.4413	25502341
*SP*1	6667	0.2215	0.0690	1.2031	22158040
*GRB*2	2885	0.3315	0.1051	1.0874	26693065, 27449805
*PPP1CA*	5499	0.2363	0.0686	0.8904	29285244
*POT*1	25913	0.3106	0.0485	0.7578	19285750
*ESR*1	2099	0.0278	0.0013	0.7493	11929836, 16033821
*MYC*	4609	0.3150	0.0947	0.7396	22941188, 28089889
*CDK*2	1017	0.3184	0.1035	0.7185	25301183
*EGFR*	1956	0.0841	0.0091	0.6921	8391303, 10767376

Additionally, we questioned whether *RS* would reflect gene risk better than |log_2_*FC|*. First, we downloaded cancer causal genes (CCGs) from the Cancer Gene Census (CGC) [60]. We obtained CCGs for four cancer types (COAD, LIHC, LUAD, and OV) separately (Additional file 1: Tables S2 and S5). Then, we obtained two gene expression data sets for each of these cancer types from two independent sources (TCGA and GEO, Additional file 1: Table S2) and performed differential expression analysis. For each data set, we ranked genes according to their |log_2_*FC|* and *RS* from high to low, separately. Next, we evaluated if CCGs were located in the top of the rank list by the GSEA method [11]. The results showed that CCGs were significantly located in the top of the *RS* rank list for all 8 data sets at a significance level of *FDR* <0.1, whereas all |log_2_*FC|*-based *FDR*s were >0.1 (Additional file 2: Fig. S2 and Additional file 1: Table S6). Here, the CCGs with low |log_2_*FC*| achieved high *RS* through crosstalk with those high |log_2_*FC*| genes in GPCM. These results indicated that *RS* was a better index for identifying casual genes, and thus, pathways enriched with high *RS* genes are likely to have important roles. Moreover, the proportion of risk genes in the top 100 of the *RS* rank list with |log_2_*FC*| <1 varied from 17 to 60% for eight data sets. This set of high-risk genes would have been overlooked if only considering the DEG analysis (Additional file 2: Fig. S3).

Finally, we calculated a pathway enrichment score, *PS*, by integrating the *RS* of all nodes in the pathway. We took the average of the *RS* values in a pathway *k* as *PS*_*k*_. By permutation, we identified the significant dysregulated pathways (details in the “Methods” section).

### Parameter optimization and improved performance compared to existing tools

**Fig. 4:**
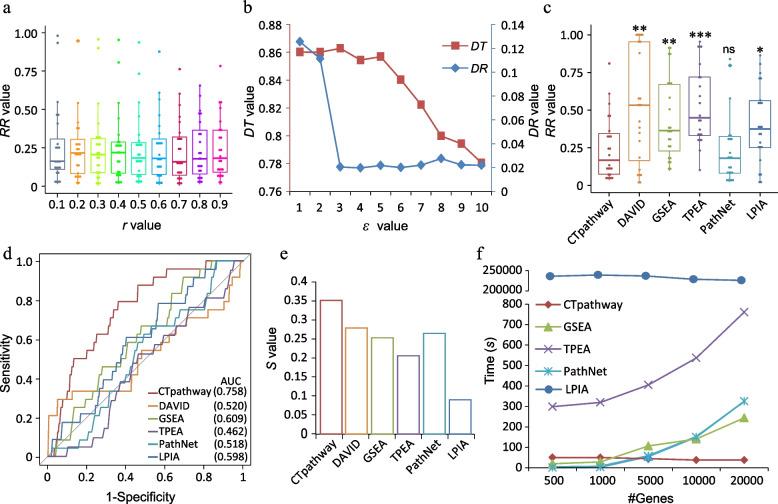
CTpathway outperforms other methods. **a** Box plot of target pathway *RR* values for different *r* values. **b** The impact of different *ε* values (*x*-axis) on *DT* (left *y*-axis) and *DR* (right *y*-axis) values. **c**–**f** Comparative analysis of the performance of different methods in terms of accuracy (*RR* and ROC curve), reproducibility, and running time, respectively. “*” represents two-sided *t*-test *P*-value < 0.05; “**” represents *P*-value < 0.01; “***” represents *P*-value < 0.001; ns represents not significant

We tested the performance of different *r* values based on 24 gold standard data sets involving 12 human diseases (Additional file 1: Table S2). In general, there was a slight variance on the performance with different *r* values. In this study, *r* was set as 0.7 because CTpathway had the best performance (Fig. 4a). In addition, we only kept *ε* digits and set values smaller than 10^−*ε*^ to 0 for the *C* matrix to improve running speed. Here, the threshold *ε* was set to 3, which consequently completes the job in less than 50 s (86.3% reduction of running time) without compromising the quality of the results (rank difference = 0.018) (Fig. 4b).

To illustrate the effectiveness of the proposed method in identifying dysregulated pathways, our results were compared with five widely used tools, including DAVID (first-generation method) [4], GSEA (second-generation method) [11], TPEA (third-generation method) [12], LPIA (fourth-generation method) [17], and PathNet (fourth-generation method) [18]. Because most of these methods only focused on the pathways defined in KEGG, we used KEGG pathways for this comparative analysis (details in the “Methods” section).

First, accuracy was compared by using 24 gold standard data sets (Additional file 1: Table S2) [36]. We compared the *RR* values of the target pathways obtained from different tools (Fig. 4c). CTpathway had the significantly lower *RR* values than other methods (no significant change compared to PathNet), indicating that our method was more accurate. Moreover, the comparisons of ROC curves and AUC values also indicated CTpathway had the best performance (Fig. 4d).

Reproducibility is also very important. Currently, most of the PEA methods are not sufficiently reproducible because of only using DEGs and insufficiently using pathway topology and molecular interaction information. To evaluate the stability of the methods, we calculated the *S* value (details in the “Methods” section) for four cancer types (COAD, LIHC, LUAD, and OV) based on different sources (TCGA RNA-seq data and GEO microarray data) of eight gene expression data sets (Additional file 1: Table S2). The results showed that CTpathway achieved the highest *S* value in all comparisons (Fig. 4e). Therefore, our data showed that CTpathway outperformed other tools in generating reproducible results.

Next, we compared the running time of CTpathway with other methods such as GSEA, TPEA, PathNet, and LPIA. Because DAVID was used on the web server, the running time of which might be interfered by the internet connection speed, it was excluded. We used simulated data sets of 500, 1000, 5000, 10,000, and 20,000 genes. Our results demonstrated that CTpathway outperformed other methods, particularly as gene number increased. As the number of genes rose, increased running time was observed in TPEA, PathNet, and GSEA, whereas no change in running time occurred in CTpathway and LPIA (Fig. 4f). However, LPIA running time was days compared to CTpathway, which took less than 50 s to analyze one set of data regardless of gene number, demonstrating that our method was independent of gene set size. Taken together, our data show that CTpathway has greater accuracy, higher reproducibility, and less running time compared to other methods.

### CTpathway identifies risk pathways in cancers

**Fig. 5:**
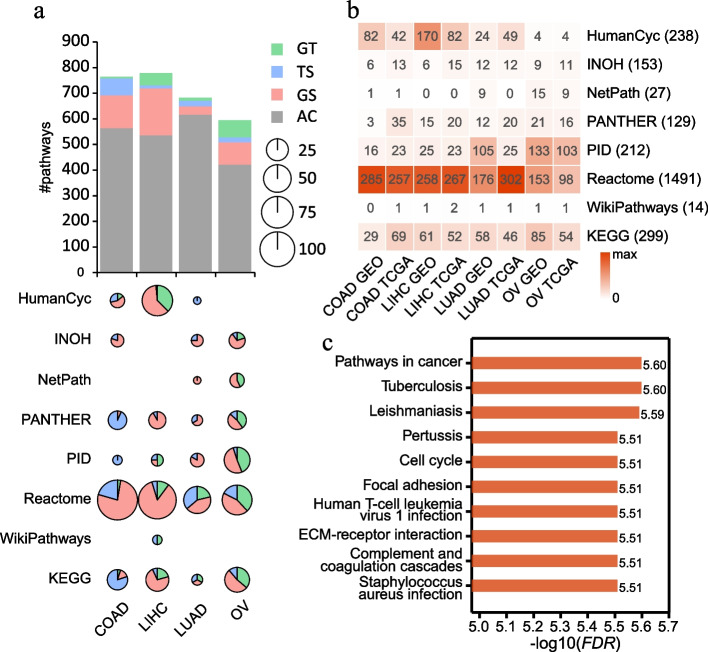
CTpathway accurately identifies well-known cancer risk pathways. **a** The comparison of identified pathways in four cancers for eight data sets. Gray color represents identified pathways across multiple cancer types (AC). Other colors represent cancer type-specific identified pathways. GT represents both GEO and TCGA data sets; TS and GS represent TCGA specific and GEO specific, respectively. For cancer type-specific identified pathways, the proportion of pathways for different data sets is shown as pie charts. **b** Heatmap of pathways identified for eight cancer data sets by CTpathway in eight pathway databases. The number of candidate pathways for each database is indicated in parentheses. **c** The bar graph shows the top 10 KEGG pathways

To demonstrate the utility of CTpathway, we firstly applied it to eight gene expression data sets of four tumor types (COAD, LIHC, LUAD, and OV; Additional file 1: Table S2). In eight pathway databases, we identified significant pathways for the four tumor types at a significance level of *FDR* < 0.01 (Fig. 5a and b, and Additional file 1: Table S7-S14). The number of identified pathways in different pathway databases differed. In general, the number of identified pathways in the Reactome database was relatively higher because of more candidate pathways. The total number of significant pathways for the eight gene expression data varied from approximately 300 to 500, accounting for 11.7~19.5% of all candidate pathways (Fig. 5b). Some well-known cancer pathways were significant in more than one cancer type (Fig. 5a and Additional file 1: Table S7-S14). For example, the “AP-1 transcription factor network” was identified as a significant pathway across four cancer types over all eight data sets. The AP-1 transcription factor is involved in a wide range of biological processes, such as cell growth, proliferation, differentiation, apoptosis, migration, and invasion [61–64]. “FOXM1 transcription factor network,” “Degradation of the extracellular matrix,” and “Activation of matrix metalloproteinases” appeared in seven data sets. Many previous studies have demonstrated that these pathways are altered in multiple cancer types, indicating their pan-cancer regulation potential [65–70]. We also identified pathways unique to a single cancer. For example, transport-related pathways (“Transferrin endocytosis and recycling” and “Passive transport by Aquaporins”) were reproducibly identified in COAD in both the GSE100179 [40] and TCGA patient cohort and not in other cancers. Many metabolism-linked pathways (“Pyruvate metabolism,” “Glycerolipid metabolism,” “Glycogen degradation II,” “Acetate conversion to acetyl-CoA,” and “Caffeine metabolism”) were specifically identified in LIHC patient cohorts in TCGA and GEO (GSE101685 [41]). Previous reports demonstrated that a large number of metabolic processes are dysregulated in LIHC to fuel tumorigenesis [71], suggesting our method accurately identifies dysregulated pathways in cancer. Several transcription or signal transduction-related pathways (“RUNX1 regulates transcription of genes involved in differentiation of HSCs,” “NOTCH1 Intracellular Domain Regulates Transcription,” “Constitutive Signaling by NOTCH1 PEST Domain Mutants”) were shared by GEO (GSE116959 [42]) and TCGA LUAD patient cohorts, but not in other cancers. In OV, immune and EMT/migration/invasion-related pathways were observed, such as “TCR,” “IL3,” “E-cadherin signaling in the nascent adherens junction,” “RUNX2 regulates genes involved in cell migration,” “Adherens junction,” and “Stabilization and expansion of the E-cadherin adherens junction” pathways. Importantly, these pathways have been reported to impact cell and/or organ functions and/or tumorigenesis [72–82]. Collectively, our results show that CTpathway accurately identifies well-known cancer risk pathways.

**Fig. 6:**
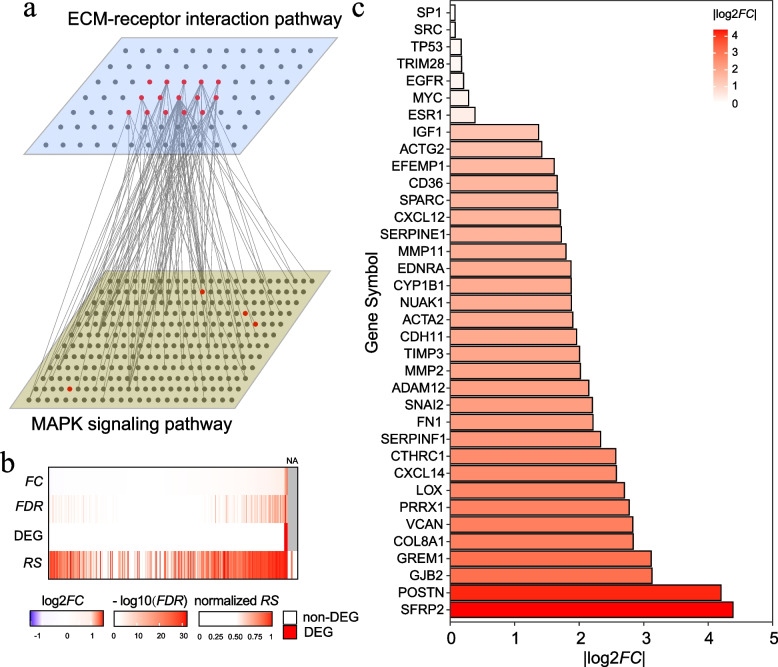
“MAPK signaling pathway” aid in EMT in OV. **a** Crosstalk between “MAPK signaling pathway” and “ECM-receptor interaction.” The red nodes represent DEGs and the gray nodes are non-DEGs. Fourteen of 15 DEGs in the “ECM-receptor interaction” pathway have a direct connection with the “MAPK signaling pathway.” **b** The risks of genes in the “MAPK signaling pathway.” Risks were measured by *FC*, *FDR*, DEG, and *RS. ***c** The bar graph shows EMT genes in the top 100 of the *RS* rank list in OV

Most of the pathways are previously verified known risk pathways for the individual cancer types, indicating CTpathway is a highly reliable tool for prioritizing the risk pathways. Taking GEO LUAD and OV data sets as examples, all the top 10 pathways for LUAD and nine of the top 10 pathways for OV have been reported (Fig. 5c, Table 3 and Additional file 1: Table S15-S19). We also compared risk pathways identified by different methods. We determined that all of the top 10 risk pathways for both LUAD and OV in CTpathway were also identified by other methods (Table 3 and Additional file 1: Table S15-S19). However, four of the top 10 pathways for LUAD or OV were identified by only one or two existing methods (Additional file 1: Table S15-S19), such as “Tuberculosis,” “Focal adhesion,” and “ECM-receptor interaction,” which play critical roles in cancer pathogenesis or progression [83–85]. Moreover, we determined the pathways only identified by CTpathway for OV (Table 4 and Additional file 1: Table S17-S19), and most of these pathways were cancer-related such as “MAPK signaling pathway” [86, 87], “Wnt signaling pathway” [88], and “Hippo signaling pathway” [89]. We also determined that these pathways had a lower proportion of DEG (Additional file 2: Fig. S4). For example, CTpathway identified “MAPK signaling pathway” (Table 4), which has important roles in the development and survival of many cancer types including ovarian cancer [86, 87]. Also, there is a crosstalk between “MAPK signaling pathway” and “ECM-receptor interaction” (Fig. 6a), which had been demonstrated to aid in EMT/migration/invasion process [90–92]. Five of six methods including CTpathway identified “ECM-receptor interaction” as a risk pathway (Table 3); however, all of the other compared methods were unable to determine “MAPK signaling pathway” as a risk pathway in EMT in OV. Furthermore, we determined that there was a lower proportion of DEGs in the “MAPK signaling pathway” (1.4% [4/295]) than that in the “ECM-receptor interaction” pathway (18.5% [15/81]); thus, most methods will identify “ECM-receptor interaction” instead of “MAPK signaling pathway.” Because the “MAPK signaling pathway” has crosstalk with “ECM-receptor interaction,” with most DEGs (14/15) in the “ECM-receptor interaction” having a direct connection with the “MAPK signaling pathway,” most “MAPK signaling pathway” genes have high *RS* (Fig. 6a, b). Therefore, only our method identified “MAPK signaling pathway” as a risk pathway. Moreover, we determined that, in the top 100 of the *RS* rank list, there are 36 EMT genes [93], of which seven have low |log_2_*FC*| (|log_2_*FC*| < 1) (Fig. 6c and Additional file 1: Table S20). These genes were easily overlooked by other methods that only considered DEGs as risk genes. Taken together, CTpathway could identify cancer risk pathways that were identified by existing methods, and importantly, also significant pathways and risk genes that were overlooked by other methods.

**Table 3:** Top 10 significant pathways identified by CTpathway based on the GSE116959 LUAD data set

ID	Pathway name	#Node	*PS*	*P*-value	*FDR*	*D*^a^	*G*^a^	*G*^a^	*P*^a^	*L*^a^
hsa05200	Pathways in cancer	454	0.187	1.37×10^−8^	2.52×10^−6^	√		√	√	
hsa05152	Tuberculosis	175	0.164	1.69×10^−8^	2.52×10^−6^		√			√
hsa05140	Leishmaniasis	72	0.233	2.58×10^−8^	2.57×10^−6^	√	√	√	√	√
hsa05133	Pertussis	52	0.239	1.03×10^−7^	3.09×10^−6^	√	√	√		√
hsa04110	Cell cycle	124	0.237	6.88×10^−8^	3.09×10^−6^	√	√	√	√	
hsa04510	Focal adhesion	199	0.201	4.49×10^−8^	3.09×10^−6^	√				
hsa05166	Human T-cell leukemia virus 1 infection	233	0.196	9.77×10^−8^	3.09×10^−6^	√				√
hsa04512	Ascorbate and ECM-receptor interaction	81	0.181	8.56×10^−8^	3.09×10^−6^	√				√
hsa04610	Complement and coagulation cascades	55	0.171	8.71×10^−8^	3.09×10^−6^	√		√	√	√
hsa05150	*Staphylococcus aureus* infection	39	0.171	8.20×10^−8^	3.09×10^−6^	√	√			√

^a^*D* DAVID, *G* GSEA, *T* TPEA, *P* PathNet, *L* LPIA

**Table 4:** Top 10 significant pathways only identified by CTpathway based on the GSE9891 OV data set

ID	Pathway name	#Node	*PS*	*P*-value	*FDR*	DEG proportion
hsa04072	Phospholipase D signaling pathway	118	0.096	1.44×10^−7^	1.65×10^−6^	0.025
hsa04010	MAPK signaling pathway	295	0.085	3.14×10^−7^	2.47×10^−6^	0.014
hsa04550	Signaling pathways regulating pluripotency of stem cells	109	0.109	7.30×10^−7^	5.08×10^−6^	0.037
hsa04310	Wnt signaling pathway	144	0.096	1.45×10^−6^	8.85×10^−6^	0.028
hsa05202	Transcriptional misregulation in cancer	19	0.140	1.85×10^−6^	1.11×10^−5^	0.053
hsa05212	Pancreatic cancer	75	0.126	2.60×10^−6^	1.49×10^−5^	0
hsa01521	EGFR tyrosine kinase inhibitor resistance	79	0.113	4.28×10^−6^	2.35×10^−5^	0.038
hsa05225	Hepatocellular carcinoma	168	0.093	9.76×10^−6^	5.12×10^−5^	0.006
hsa04390	Hippo signaling pathway	153	0.090	1.15×10^−5^	5.85×10^−5^	0.032
hsa05224	Breast cancer	145	0.096	3.42×10^−5^	1.49×10^−4^	0.014

### CTpathway identifies risk pathways in data sets with fewer DEGs

**Fig. 7:**
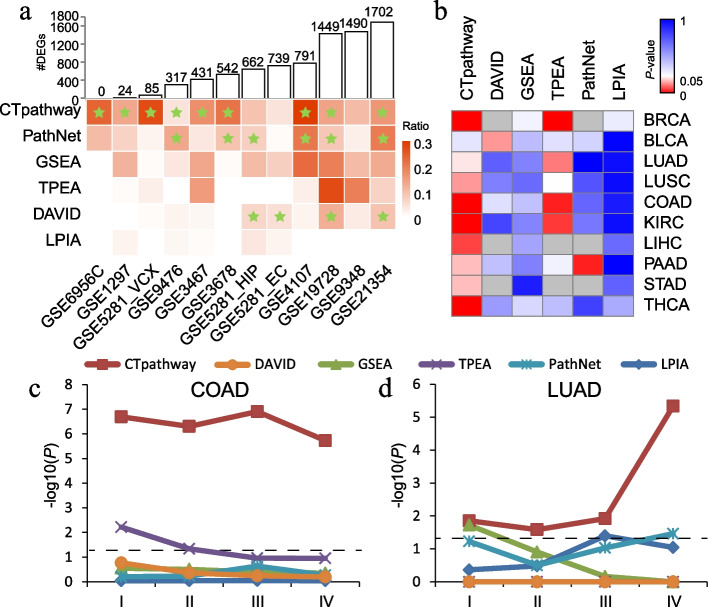
CTpathway identifies significant pathways in data sets with few DEGs and early-stage cancer patients. **a** The comparison of identified pathways for data sets with a different number of DEGs in six methods. The bar graph shows the number of DEGs for 12 representative data sets. The heatmap shows the number of significant pathways at the significance level of *FDR* < 0.05, identified by different methods for data sets with a different number of DEGs, divided by the number of all candidate pathways. The target pathways are marked as green stars. **b** The heatmap of enrichment results (*P*-value) for the target pathways of 10 early-stage cancer types using different methods. **c**, **d** Enrichment results (*P*-value, *y*-axis) for the target pathways of TCGA COAD data sets of tissue samples (**c**) and LUAD blood samples (**d**) of different stages using different methods. The dashed line represents a cutoff (*P*-value = 0.05)

Because of its algorithmic properties, we postulated that CTpathway would be useful for data sets with a small number of DEGs. To test this, we screened DEGs for 24 gold standard data sets at a level of |log_2_*FC*| > 1 and *FDR* < 0.1 and selected 12 representative gold standard data sets with different numbers of DEGs ranging from 0 to 1702 (Fig. 7a and Additional file 1: Table S3). We compared KEGG pathways identified by CTpathway with those by other five methods (DAVID, GSEA, TPEA, PathNe, and LPIA) at a significance level of *FDR*-corrected *P*-value < 0.05 (Fig. 7a). The number of significant pathways identified by CTpathway was independent from the number of DEGs. For data sets with fewer DEGs, CTpathway could identify more pathways than all other methods. However, other methods, including DAVID, GSEA, and TPEA, showed a greater dependency on the number of DEGs. They could only identify a small number of significant pathways for data sets with fewer DEGs (e.g., GSE6956C [94] and GSE1297 [95]). Furthermore, CTpathway could identify target pathways for most (9/12) of the data sets, whereas other methods had a lower rate of identification and overlooked them, especially for data sets with fewer DEGs. We also compared significant pathways at a level of nominal *P*-value < 0.05 (Additional file 2: Fig. S5), and CTpathway could identify target pathways independent on the number of DEGs. These results demonstrated that CTpathway outperformed other methods for target pathway identification, particularly when there are a small number of DEGs.

### CTpathway identifies risk pathways in early-stage cancer

Cancer diagnosis relies on detecting symptoms followed by histology/pathology evaluation. Identification of altered pathways indicative of pre-malignancy or early-stage cancer is critical for disease prevention and earlier treatment, leading to improved outcomes for patients. Early-stage cancers usually show smaller changes at the molecular level than late-stage cancers. We tested whether CTpathway could identify risk pathways for early-stage disease in cancer patients. First, samples of 10 cancer types that included stages I, II, III, and IV were obtained from TCGA. We selected the KEGG annotated ten pathways specific for the ten cancer types (Additional file 1: Table S2). For each cancer type of each stage, pathway enrichment analysis was performed by CTpathway and other methods. The *P*-values of target pathways were compared by different methods. The results showed that CTpathway performed better than the other methods for tissue samples (Fig. 7b, c and Additional file 2: Fig. S6). In general, the CTpathway *P*-values of target pathways were smaller than those of other methods. Even in the early-stage (stage I) patients, 9/10 target pathways of cancer types could be identified by CTpathway at a significance level of *P*-value < 0.05, whereas all but one other method could either not identify any or only identified one target pathway in early-stage patients for one cancer type (Fig. 7b). In addition, we also evaluated blood samples, which are easier to obtain from patients as compared to tissue samples. CTpathway was applied to the data sets of the blood samples (GSE20189 [44]) from LUAD patients of different stages (I, II, III, and IV), and it identified the target pathway in the early stage as well as performed better than the other methods across all cancer stages (Fig. 7d). These results demonstrate that CTpathway may be useful for early disease diagnosis.

### CTpathway identifies cell type-related pathways in scRNA-seq data

**Fig. 8:**
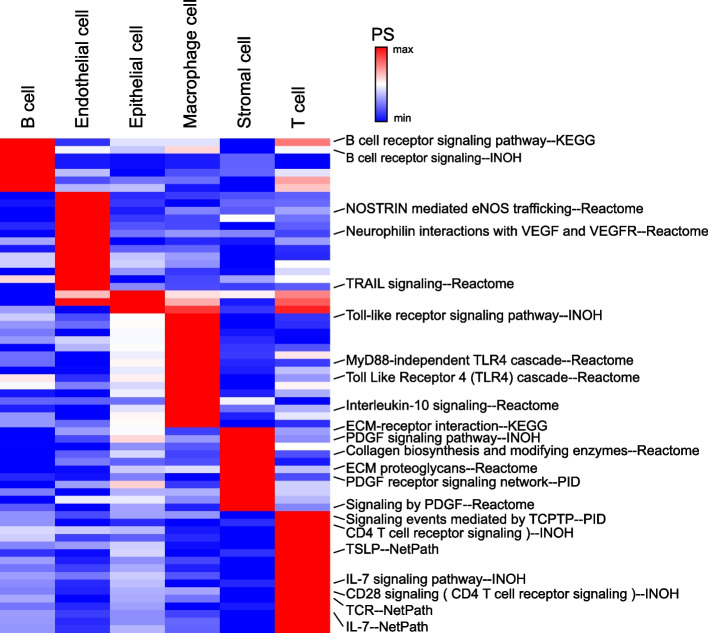
CTpathway identifies cell type-related pathways in BRCA scRNA-seq data. The heatmap of enrichment results (*PS* value) of pathways in each cell type determined by CTpathway

Due to characteristics of scRNA-seq data such as drop-out events and low library sizes, the number of DEGs for a subgroup or cell type is typically low. Because CTpathway is not limited by DEG number, we postulated it could be utilized in scRNA-seq data. To test this, we obtained BRCA scRNA-seq data (GSE118389 [45]). Cell types were annotated (B cell, T cell, macrophage, endothelial cell, epithelial cell, and stromal cell) according to the reported study [45]. Differential expression analysis was performed between one cell type and the others by Seurat V3.2.2 [49]. Then, CTpathway was applied to each cell type. The pathway enrichment results showed that CTpathway could identify known cell type-related pathways in each cell type (Fig. 8). For example, “B cell receptor signaling pathway” was significant in B cell (*FDR* = 1.48×10^−6^) [96]; “Neurophilin interactions with VEGF and VEGFR” was significant in endothelial cell (*FDR* = 5.06×10^−6^) [97]; “Toll-like receptor signaling pathway” was significant in macrophage (*FDR* = 6.47×10^−6^) [98]; “ECM-receptor interaction” was significant in stromal cell (*FDR* = 1.61×10^−6^) [99]; “TCR” was significant in T cell (*FDR* = 6.21×10^−7^) [100]. Compared to other methods, CTpathway showed the lowest *RR* value for the “B cell receptor signaling pathway” in B cell (Additional file 2: Fig. S7). These results demonstrated that CTpathway could effectively identify cell type-related functions or pathways in scRNA-seq data.

### Reduction of pathway redundancy

Redundancy is a frequently neglected problem for most PEA methods. Pathways sharing genes lead to functional similarities. As a result, it is difficult to extract representative pathways from redundant information [7, 101, 102]. CTpathway automatically clusters enriched pathways into non-redundant groups. Briefly, we constructed a similarity network after obtaining significant pathways based on a particular cutoff of the *Jaccard* similarity coefficient for shared genes among all significant pathway pairs. MCL clustering algorithm [55] was employed to absorb most redundancies into representative clusters. Each cluster was renamed as the name of the most significant pathway in this cluster. Taking TCGA COAD stage I patient samples as an example (Additional file 2: Fig. S8), we determined some clusters with two or more pathways, and our method enables robust identification of the remaining single node clusters, indicating that these risk pathways reveal potentially targetable pathways, as they have the least amount of crosstalk with other pathways. Therefore, CTpathway is designed to obtain non-redundant pathway information to better interpret pathway enrichment results, and this is dictated according to the needs of the user who input a cutoff of the *Jaccard* similarity coefficient on the web server.

### Web-based implementation of CTpathway

We provided an online web tool for users to perform pathway enrichment analysis with CTpathway (Additional file 2: Fig. S9). Users can input data including gene (gene symbol or entrez ID), both log_2_*FC* and *P*-value or either. By selecting several parameters, input Email address, and clicking the “run” button (Additional file 2: Fig. S9a and b, more details in the Web Manual page), CTpathway returns enrichment results shown in the table in the result page of the web server. The results are also visualized by a bar graph, a bubble plot, and an enrichment map (Additional file 2: Fig. S9c-e). Users can choose any or all results according to their needs. In the web server, results only take a few minutes.

## Discussion

PEA is a useful method for exploring gene set function. However, most existing methods did not consider pathway crosstalk and priori knowledge. In this study, we designed and provided to the research community CTpathway, a crosstalk-based PEA method through a global pathway crosstalk map (GPCM) by using multiple sources of pathways and priori knowledge in human.

First, we collected TF-gene regulation, PPI, and gene-gene interaction and constructed a GPCM. The topological property analysis showed that the degree distribution approximately displayed a power law distribution, which was similar to most biological networks. Then, we integrated *FC* and *P*-value for each gene from differential expression analysis as gene differential expression score (*DE*). Next, we obtained a crosstalk effect matrix by the multi-RWR algorithm and calculated a final risk score (*RS*) by integrating the *DE* and crosstalk effects. By enrichment analysis of the CGC genes, we demonstrated that *RS* was a better index for identifying risk genes, and identified important genes with a high *RS* and low |log_2_*FC*| that were overlooked by other methods that relied on |log_2_*FC*|. Finally, we calculated a pathway enrichment score by averaging *RS* for genes in the pathway and identified significantly dysregulated pathways by permutation. Our optimization process reduced ~86.3% of the original running time. Furthermore, the performance of CTpathway is significantly better compared with existing methods in terms of accuracy (*RR* and AUC value), reproducibility, and running time. In addition, by applying CTpathway to cancer patient samples, we determined that CTpathway could identify critical pathways, which were not identified by other methods. For the data sets with a small number of DEGs, CTpathway was also useful and outperformed the other methods. Notably, CTpathway outperformed other methods in identifying target pathways in early-stage cancer tissues and blood samples. For scRNA-seq data, which can have small DEG numbers, CTpathway could effectively identify cell type-related pathways. Our results demonstrate that CTpathway could be applied in disease analysis, and especially for data sets with fewer DEGs, early cancer diagnosis, which may lead to starting treatment earlier, and scRNA-seq data. We also developed an online web tool to allow users to easily and freely perform PEA with CTpathway.

This study provides a new useful PEA method, CTpathway, for over 2500 pathways in eight pathway databases, and showed that CTpathway performed better than other widely used methods. We evaluated CTpathway performance using the commonly used standard data sets. However, these data sets are limiting because there are only 24 target pathways for 24 diseases, indicating a need in the field for more gold standard data sets for the evaluation of pathway enrichment analysis methods. If the data sets contained additional known risk pathways for diseases, the methods could be evaluated more precisely using the precision-recall curve and AUPRC. In addition, CTpathway still has limitations related to reproducibility, which is consistent with PEA methods overall. For example, when different data sets belonging to the same disease serve as input, the results may differ. While differences in samples and sample handling and processing between different labs contribute to reproducibility challenges, CTpathway was more reproducible than the other methods, showing ~35% overlap between different data sets tested.

Of note, the NT methods are highly dependent on the information of interactions, such as TF-gene regulations, PPIs, and gene-gene interactions, and thus, incomplete information will limit the development of these methods. In this study, TF-gene regulations come from the TRANSFAC database. Recently, several other resources of TF-gene regulation have been provided [103, 104]. Adding more TF-gene regulations might lead to a potential improvement of CTpathway. Notably, CTpathway could be extended to predict non-coding RNA (ncRNA) functions by adding ncRNA regulations or interactions into GPCM. Moreover, CTpathway only focuses on *Homo sapiens* in this version. Through constructing GPCM for other species, CTpathway could be used to identify risk pathways of other species. Although future studies will be needed to investigate these areas, CTpathway provides a new publicly available method that should result in new discoveries in multiple fields of biology and disease research.

## Conclusions

This study presents a novel pathway crosstalk-based method, CTpathway, for performing pathway enrichment analysis. CTpathway outperformed existing methods on accuracy, reproducibility, and speed. CTpathway exclusively identified critical pathways in several cancer types. Furthermore, CTpathway was useful even for data sets with few differentially expressed genes and could identify target pathways in early-stage cancer patient samples, which could lead to earlier treatment, and identify cell type-related pathways for scRNA-seq data. Finally, we provide an interactive and easy-to-use web server so users can conveniently perform pathway enrichment analysis and discover disease-risk pathways.

## Supplementary Information

**Additional file 1: Table S1.** Information of protein-protein (PPI) included in this study. **Table S2.** Summary of data sets analyzed in this study. **Table S3.** Summary of 12 gold standard data sets. **Table S4.** The degree of genes in the GPCM. **Table S5.** List of CGC genes of four cancer types. **Table S6.** GSEA enrichment results of *RS* or |log_2_*FC*| for CGC genes. **Tables S7-S14.** Significant (*FDR* < 0.01) pathways identified by CTpathway for GEO COAD, TCGA COAD, GEO LIHC, TCGA LIHC, GEO LUAD, TCGA LUAD, GEO OV, TCGA OV. **Tables S15-S18.** Comparative results show significant KEGG pathways identified by CTpathway with other methods from GEO LUAD, TCGA LUAD, GEO OV, TCGA OV data. **Table S19.** Significant (*FDR* < 0.01) KEGG pathways for GEO OV data only identified by CTpathway. **Table S20.** EMT genes in the top 100 of *RS* rank list.

**Additional file 2: Figure S1.** Crosstalk effect consideration detects genes known to be associated with LUAD. **Figure S2.** CGC genes are in top of the *RS* rank list, but not top of the |log_2_*FC*| rank list. **Figure S3.** The proportion of risk genes, overlooked by other methods. **Figure S4.** Comparison of DEG proportions between pathways specifically identified by CTpathway and non-specific pathways. **Figure S5.** Significant pathways in data sets with a small number of DEGs, identified by CTpathway. **Figure S6.** Comparison of enrichment result (*P*-value) of target pathways for TCGA cancer stages (I, II, III and IV), obtained by different methods. **Figure S7.** Enrichment result (*RR* value) comparison of target pathways for B cell by different methods. **Figure S8.** An enrichment map constructed from early-stage COAD data set. **Figure S9.** The publicly available CTpathway web tool.

## Data Availability

The CTpathway web server is available from http://www.jianglab.cn/CTpathway/. The CTpathway stand-alone program is available from https://github.com/Bioccjw/CTpathway [29]. All data used could be obtained from public sources (details in Additional file 1: Table S2). Twenty-four gold standard data sets were obtained from the KEGGdzPathwaysGEO R package [36]. Eight gene expression data of four cancer types (COAD, LIHC, LUAD, and OV) were downloaded from the GEO database [38] (https://www.ncbi.nlm.nih.gov/geo/) under accession numbers GSE100179 [40], GSE101685 [41], GSE116959 [42], and GSE9891 [43] and the TCGA database [39] (https://portal.gdc.cancer.gov/). Cancer data of different stages were downloaded from the TCGA (https://portal.gdc.cancer.gov/) and GEO (https://www.ncbi.nlm.nih.gov/geo/, under accession number GSE20189 [44]) databases. BRCA scRNA-seq data were obtained from the GEO database (https://www.ncbi.nlm.nih.gov/geo/) under accession number GSE118389 [45]. Cancer causal genes were obtained from the Cancer Gene Census (CGC) [60] (https://cancer.sanger.ac.uk/census/).

## Abbreviations

BRCA: Breast cancer
C: Crosstalk effect matrix
COAD: Colon adenocarcinoma
DE: Differential expression score
DEGs: Differentially expressed genes
DR: Rank difference
DT: Time difference
ESs: Enrichment scores
FC: Fold change
FCS: Functional class scoring
FDR: False discovery rate
GEO: Gene Expression Omnibus
GO: Gene Ontology
GPCM: Global pathway crosstalk map
GPD: Generalized Pareto distribution
GSEA: Gene set enrichment analysis
KEGG: Kyoto Encyclopedia of Genes and Genomes
LIHC: LIVER hepatocellular carcinoma
LPIA: Latent pathway identification analysis
LUAD: Lung adenocarcinoma
MCL: Markov Cluster
multi-RWR: Multiple random walk with restart
NT: Network topology-based
ORA: Over-representation analysis
PathNet: Pathways based on network information
PEA: Pathway enrichment analysis
PPI: Protein-protein interaction
PS: Pathway enrichment score
PT: Pathway topology-based
RR: Rank ratio
RS: Risk score
S: Stability
scRNA-seq: Single-cell RNA-seq
TCGA: The Cancer Genome Atlas
TF: Transcription factor
THCA: Thyroid cancer
