# Supplementary material for: CTpathway: a CrossTalk-based pathway enrichment analysis method for cancer research
Source: Genome Med. 2022 Oct 13;14:118. doi: 10.1186/s13073-022-01119-6 (PMC9563764; doi:10.1186/s13073-022-01119-6)
Supplement: Supplementary file 2 — Additional file 2: Figure S1. Crosstalk effect consideration detects genes known to be associated with LUAD. Figure S2. CGC genes are in top of the RS rank list, but not top of the |log2FC| rank list. Figure S3. The proportion of risk genes, overlooked by other methods. Figure S4. Comparison of DEG proportions between pathways specifically identified by CTpathway and non-specific pathways. Figure S5. Significant pathways in data sets with a small number of DEGs, identified by CTpathway. Figure S6. Comparison of enrichment result (P-value) of target pathways for TCGA cancer stages (I, II, III and IV), obtained by different methods. Figure S7. Enrichment result (RR value) comparison of target pathways for B cell by different methods. Figure S8. An enrichment map constructed from early-stage COAD data set. Figure S9. The publicly available CTpathway web tool. [file 13073_2022_1119_MOESM2_ESM.pdf]

Additional file 2

CTpathway: a CrossTalk-based pathway enrichment analysis method for cancer research

Haizhou Liu[†], Mengqin Yuan[†], Ramkrishna Mitra[†], Xu Zhou, Min Long, Wanyue Lei, Shunheng Zhou, Yu-e Huang, Fei Hou, Christine M. Eischen[*], Wei Jiang[*]

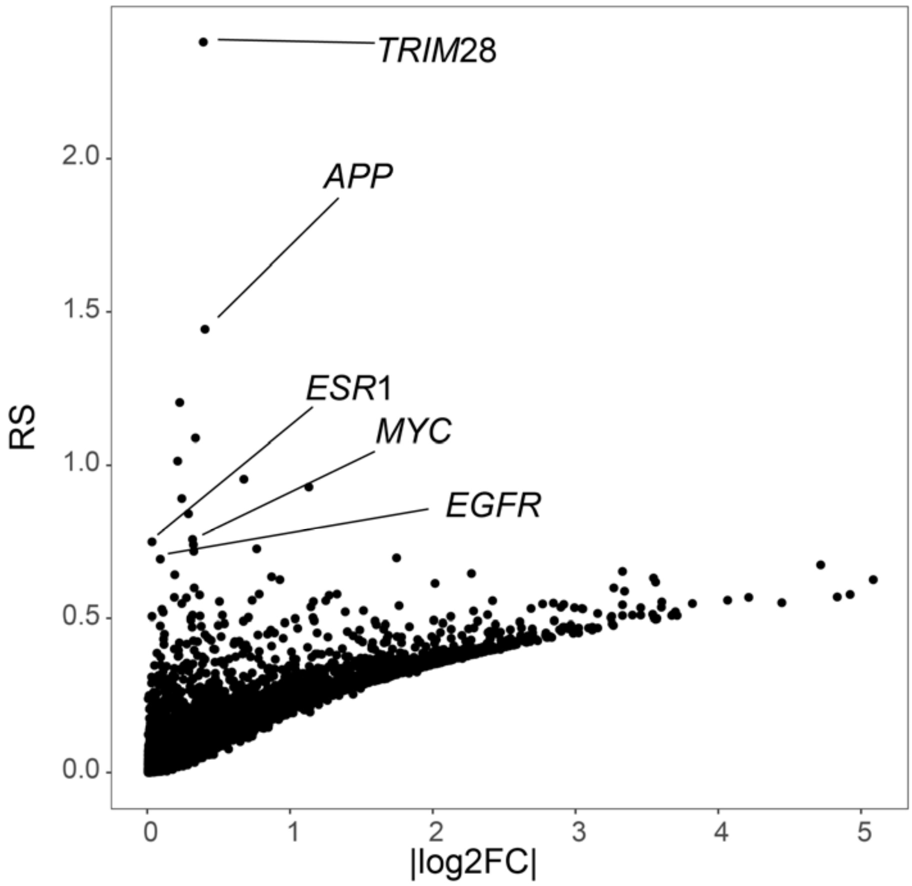

Figure S1. Crosstalk effect consideration detects genes known to be associated with LUAD. Dot plot of genes for GSE116959 LUAD data set. Examples for well-known biomarkers with high *RS* value and low $|\log_2 FC|$ value are marked.

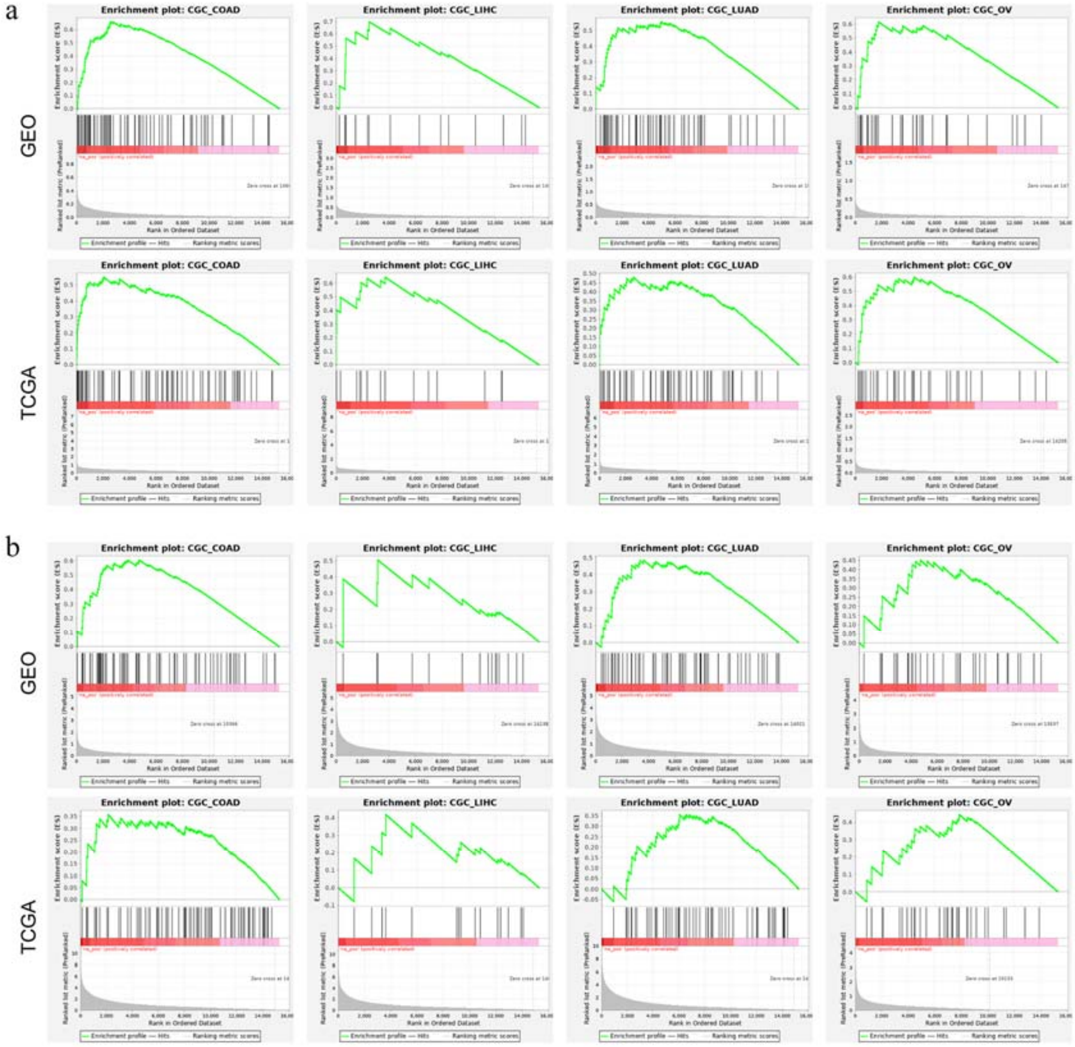

Figure S2. CGC genes are in top of the *RS* rank list, but not top of the $|\log_2 FC|$ rank list. The location of CGC genes enriched in (a) *RS* rank list and (b) $|\log_2 FC|$ rank list. Genes are ranked from high to low according to *RS* or $|\log_2 FC|$.

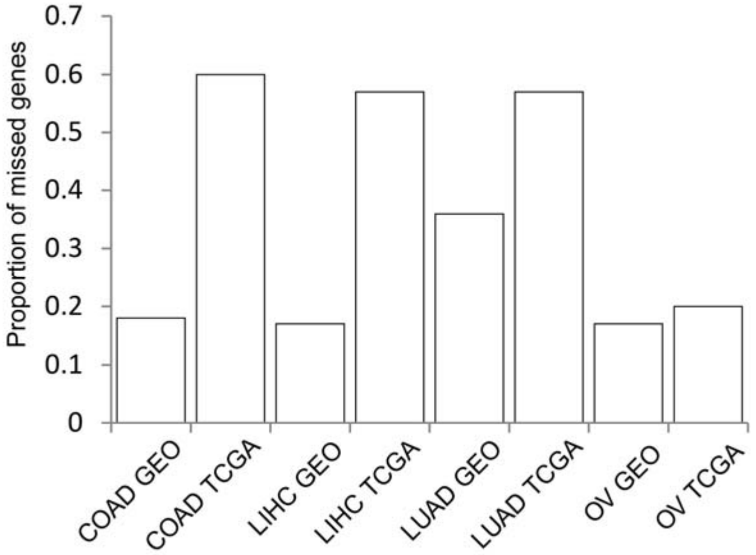

Figure S3. The proportion of risk genes, overlooked by other methods. The proportion of genes with $|\log_2 FC|<1$ in the top 100 of the *RS* rank list for each dataset.

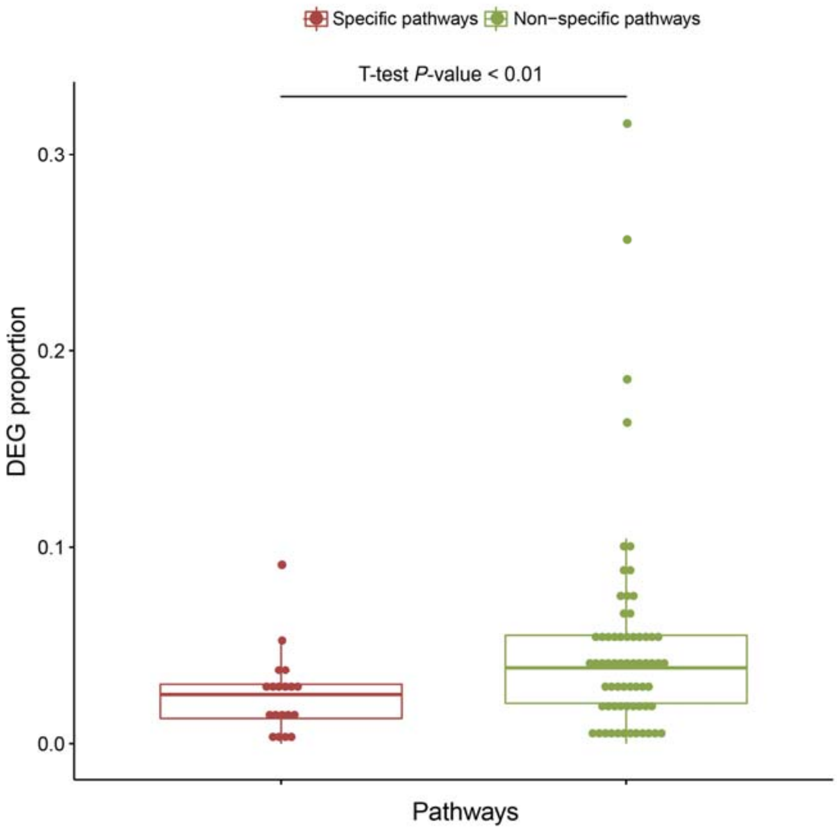

Figure S4. Comparison of DEG proportions between pathways specifically identified by CTpathway and non-specific pathways. The red dots represent specific pathways (identified only by CTpathway); the green dots represent non-specific pathways (identified by both CTpathways and the other methods). The y-axis represents the proportion of DEGs in each pathway.

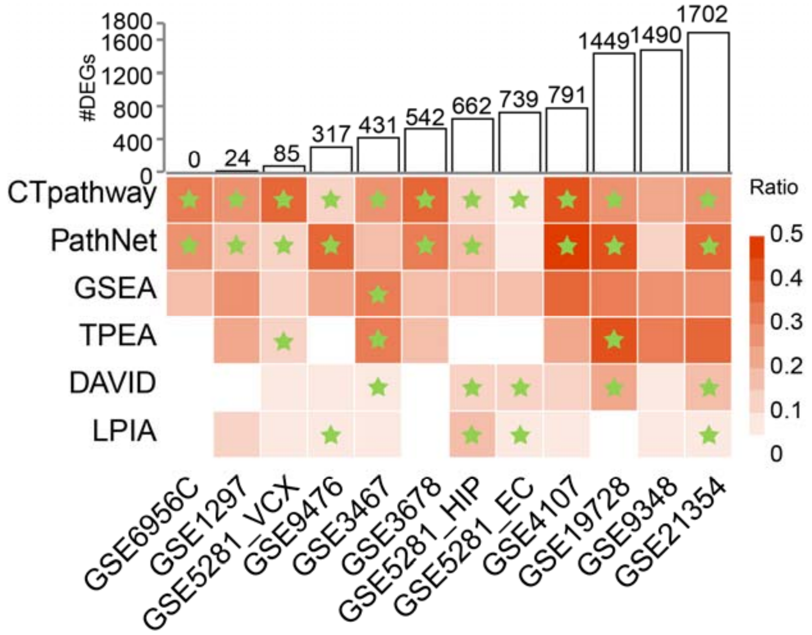

Figure S5. Significant pathways in data sets with a small number of DEGs, identified by CTpathway. The bar graph shows the number of DEGs for 12 representive data sets. The heatmap (below) shows the number of significant pathways identified by different methods at the significant level of *P*-value < 0.05 devided by the number of all candidate pathways. The target pathways are marked as green stars.

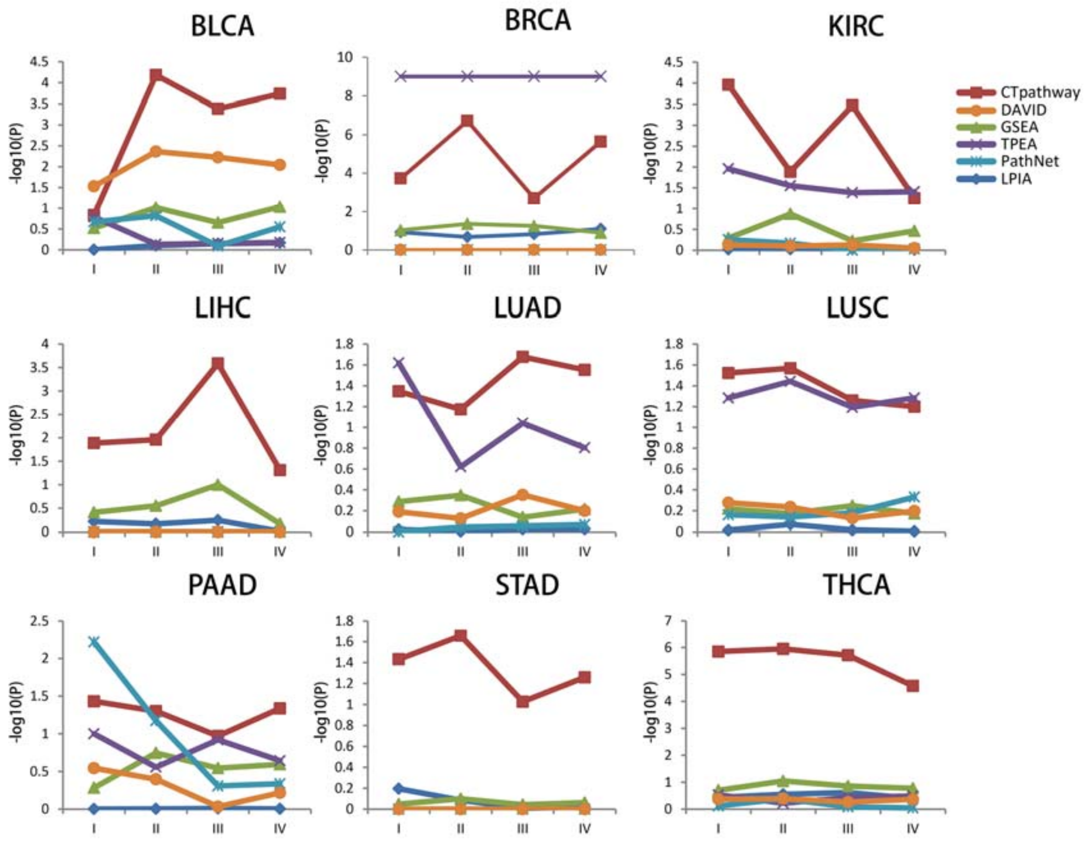

Figure S6. Comparison of enrichment result (*P*-value) of target pathways for TCGA cancer stages (I, II, III and IV), obtained by different methods.

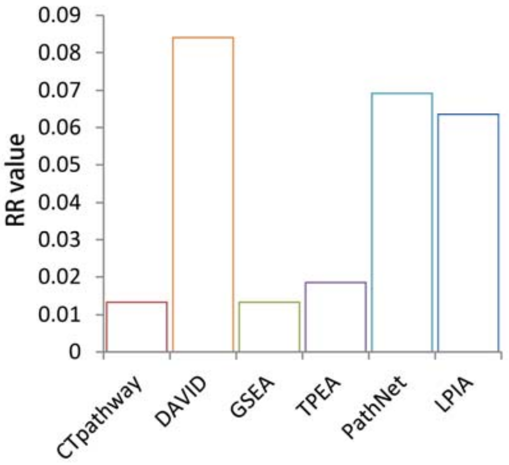

Figure S7. Enrichment result (*RR* value) comparison of target pathways for B cell by different methods.

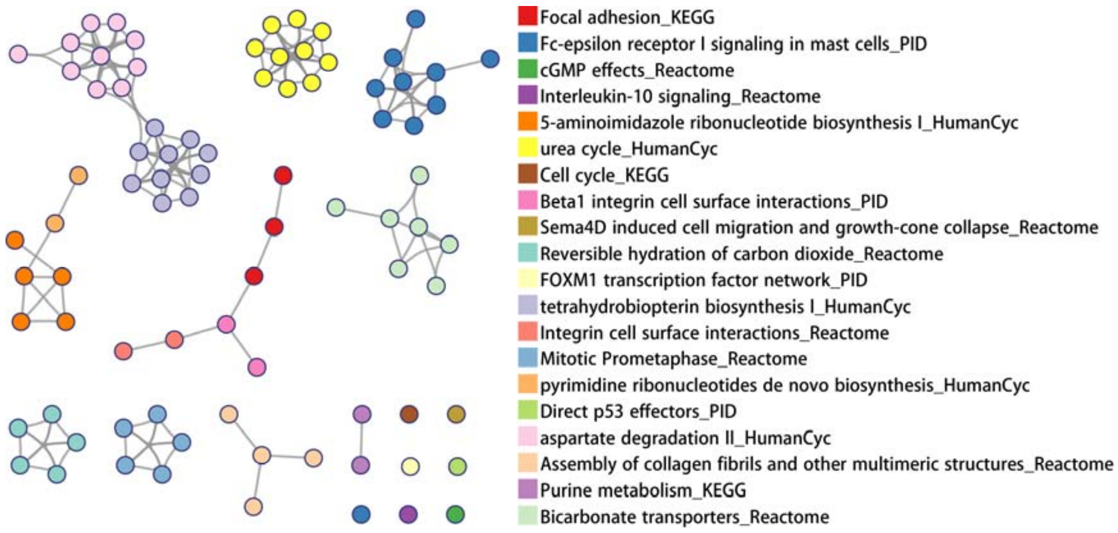

Figure S8. An enrichment map constructed from early-stage COAD data set. The top 20 non-redundant pathways or clusters with low *FDR* determined with CTpathway. For each cluster, the top 10 pathways with low *FDR* are shown in the enrichment map if more than 10 pathways were within one cluster.

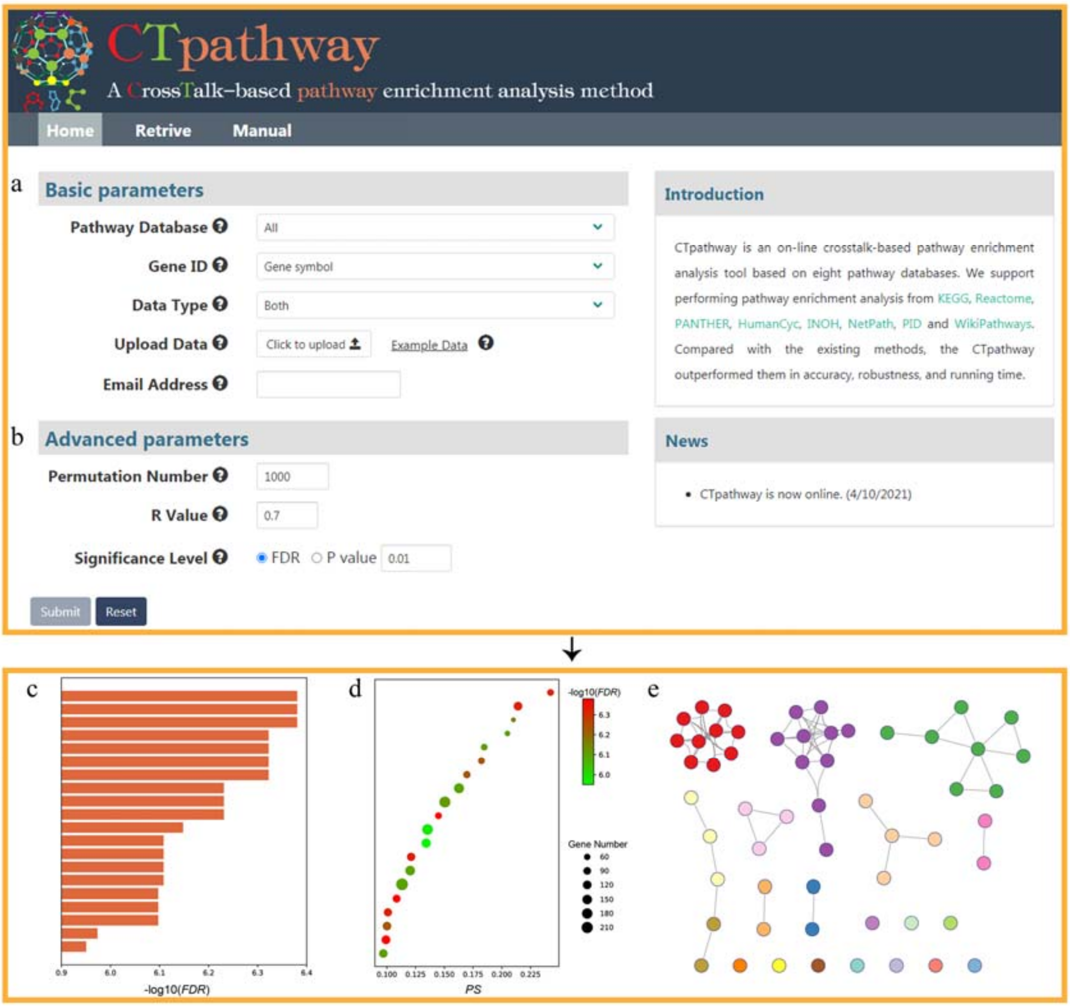

Figure S9. The publicly available CTpathway web tool. (a) Basic parameters input. (b) Advanced parameters input. (c-e) The visulized results are shown in the bar graph (c), bubble plot (d) and enrichment map (e).
